# Supplementary material for: Mutations in PCYT2 disrupt etherlipid biosynthesis and cause a complex hereditary spastic paraplegia
Source: Brain. 2019 Oct 22;142(11):3382–97. doi: 10.1093/brain/awz291 (PMC6821184; doi:10.1093/brain/awz291)
Supplement: awz291_Supplementary_Data [file awz291_supplementary_data.zip › awz291-Suppl_data/Supplementary_Data4.zip!/Supplementary information 3 - fibroblast lipidomics.zip!/Fibroblast lipidomics/Patient1 vs Patient2/Statistical plots and data/CE species/Boxplots.pdf]

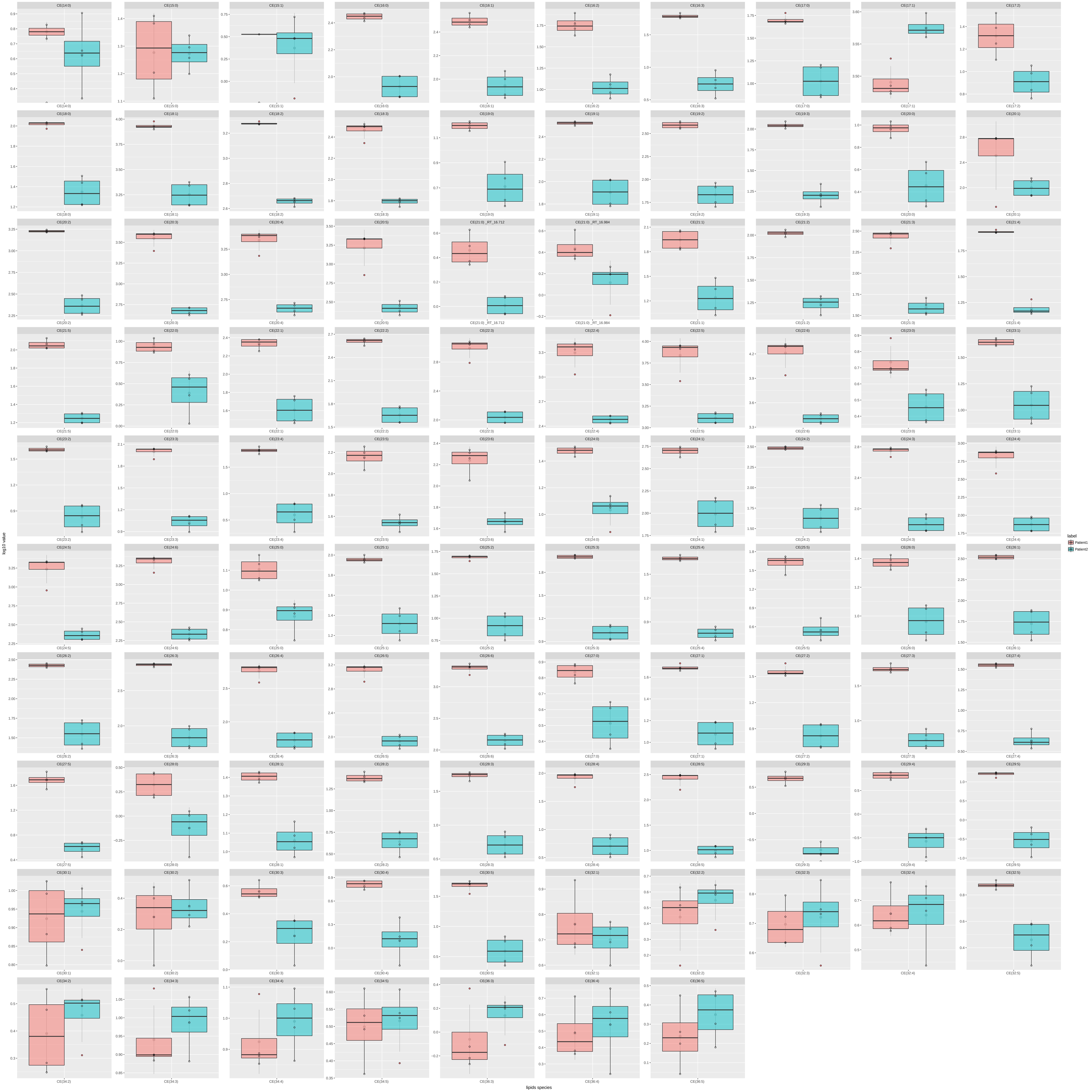
CE(14:0)
CE(15:0)
CE(15:1)
CE(16:0)
CE(16:1)
CE(16:2)
CE(16:3)
CE(17:0)
CE(17:1)
CE(17:2)
CE(18:0)
CE(18:1)
CE(18:2)
CE(18:3)
CE(19:0)
CE(19:1)
CE(19:2)
CE(19:3)
CE(20:0)
CE(20:1)
CE(20:2)
CE(20:3)
CE(20:4)
CE(20:5)
CE(21:0)_RT_16.712
CE(21:0)_RT_16.984
CE(21:1)
CE(21:2)
CE(21:3)
CE(21:4)
CE(21:5)
CE(22:0)
CE(22:1)
CE(22:2)
CE(22:3)
CE(22:4)
CE(22:5)
CE(22:6)
CE(23:0)
CE(23:1)
CE(23:2)
CE(23:3)
CE(23:4)
CE(23:5)
CE(23:6)
CE(24:0)
CE(24:1)
CE(24:2)
CE(24:3)
CE(24:4)
CE(24:5)
CE(24:6)
CE(25:0)
CE(25:1)
CE(25:2)
CE(25:3)
CE(25:4)
CE(25:5)
CE(26:0)
CE(26:1)
CE(26:2)
CE(26:3)
CE(26:4)
CE(26:5)
CE(26:6)
CE(27:0)
CE(27:1)
CE(27:2)
CE(27:3)
CE(27:4)
CE(27:5)
CE(28:0)
CE(28:1)
CE(28:2)
CE(28:3)
CE(28:4)
CE(28:5)
CE(29:3)
CE(29:4)
CE(29:5)
CE(30:1)
CE(30:2)
CE(30:3)
CE(30:4)
CE(30:5)
CE(32:1)
CE(32:2)
CE(32:3)
CE(32:4)
CE(32:5)
CE(34:2)
CE(34:3)
CE(34:4)
CE(34:5)
CE(36:3)
CE(36:4)
CE(36:5)
log10 value
lipids species
label
Patient1
Patient2
